# Mechanisms of Hydroxyurea-Induced Cellular Senescence: An Oxidative Stress Connection?

**DOI:** 10.1155/2021/7753857

**Published:** 2021-10-18

**Authors:** Sunčica Kapor, Vladan Čokić, Juan F. Santibanez

**Affiliations:** ^1^Department of Hematology, Clinical Hospital Center “Dr. Dragisa Misovic-Dedinje”, University of Belgrade, Serbia; ^2^Molecular Oncology Group, Institute for Medical Research, National Institute of Republic of Serbia, University of Belgrade, Belgrade, Serbia; ^3^Centro Integrativo de Biología y Química Aplicada (CIBQA), Universidad Bernardo O'Higgins, Santiago, Chile

## Abstract

Hydroxyurea (HU) is a water-soluble antiproliferative agent used for decades in neoplastic and nonneoplastic conditions. HU is considered an essential medicine because of its cytoreduction functions. HU is an antimetabolite that inhibits ribonucleotide reductase, which causes a depletion of the deoxyribonucleotide pool and dramatically reduces cell proliferation. The proliferation arrest, depending on drug concentration and exposure, may promote a cellular senescence phenotype associated with cancer cell therapy resistance and inflammation, influencing neighboring cell functions, immunosuppression, and potential cancer relapse. HU can induce cellular senescence in both healthy and transformed cells in vitro, in part, because of increased reactive oxygen species (ROS). Here, we analyze the main molecular mechanisms involved in cytotoxic/genotoxic HU function, the potential to increase intracellular ROS levels, and the principal features of cellular senescence induction. Understanding the mechanisms involved in HU's ability to induce cellular senescence may help to improve current chemotherapy strategies and control undesirable treatment effects in cancer patients and other diseases.

## 1. Introduction

Hydroxyurea (HU), also called hydroxycarbamide, is a simple hydroxylated compound with the molecular formula CH_4_N_2_O_2_, structurally an analog of urea and initially synthesized in 1869 [1–4]. Although HU can exist in two tautomeric forms, the drug primarily adopts the keto form due to its significantly higher stability than the imino form. Moreover, HU is a weak acid containing three ionizable protons, with a pKa of 10.6 [5].

HU is a nonalkylating antineoplastic agent used for hematological malignancies, infectious diseases, and dermatology [6]. The first evidence of its antineoplastic effects was obtained in the late 1950s in experiments conducted on L1210 leukemia cells and solid tumors [7]. In the 1960s, clinical trials demonstrated the drug's efficacy mainly against myeloproliferative disorders [2, 3].

HU has an acceptable short-term toxicity profile in most patients and is currently used as the first-line of chemotherapy in hematological malignancies such as myeloproliferative neoplasm (MPN) characterized by a mutation in Janus kinase 2 (J*AK2*), calreticulin (*CALR*), and myeloproliferative leukemia virus oncogene (MPL) genes [8–11]. Also, this agent is indicated to treat sickle-cell anemia, HIV infection, and thrombocythemia [2, 3, 12]. Moreover, it is effective for the management of refractory psoriasis, likely due to inhibition of epithelial proliferation, thus restoring the typical appearance of the patient's thickened epidermis [13–15]. In addition, HU has been used as a palliative treatment for acute myelogenous leukemia in elderly patients unfit for intensive chemotherapy [16]. Because of its positive effects of therapy, this drug is defined as an “essential medicine” by the World Health Organization [17].

## 2. Mechanisms of the Inhibition of Cell Proliferation by Hydroxyurea

**Figure 1:**
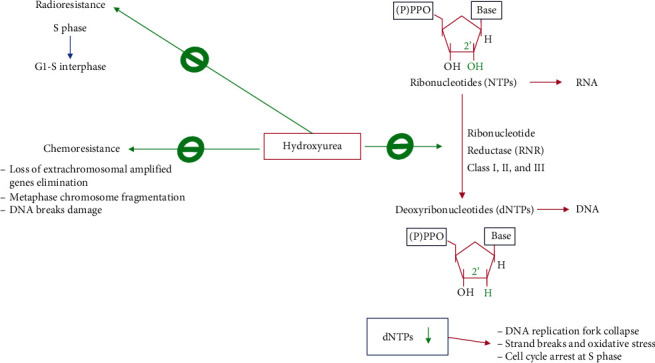
Main mechanisms of hydroxyurea cytotoxicity. HU functions as a radiation sensitizer by synchronizing cancer cells in the radiation-sensitive cell G1-S cycle interphase and inhibition of the DNA damage repair response. Also, HU sensitizes cancer cells to chemotherapy by promoting loss of extrachromosomal amplified gene elimination, metaphase chromosome, and DNA breaks damage. Moreover, HU inhibits the ribonucleotide reductase (RNR) that results in a drastic reduction of the deoxyribonucleotide pool necessary for DNA synthesis. Depletion of dNTPs promotes DNA replication fork collapse, strand break, and oxidative stress. For more details, see the text.

HU functions as a radiation sensitizer because of its capacity to synchronize cancer cells in the radiation-sensitive cell cycle phase and inhibit the repair response of DNA damage produced by radiation [18]. This drug abolishes the relatively radioresistant cells at the S phase of the cell cycle, reducing highly DNA synthesizing cells and increasing the frequency of the surviving cells at the relatively radiosensitive portion (G1–S interphase) of the cell cycle (Figure 1) [19, 20]. In addition, HU radio-sensitization in patients with advanced cervical cancer increases progression-free survival in the stages III and IVA disease cohort; moreover, HU activities have been evaluated in high-grade gliomas, non-small-cell lung cancer, head and neck cancer, and cervical carcinoma with different grades of success [21].

Furthermore, HU regulates tumor cell resistance to chemotherapy because it accelerates the loss of extrachromosomal amplified genes implicated in therapy sensitivity (Figure 1) [2, 22]. Moreover, it may induce metaphase chromosome fragmentation by directly affecting DNA integrity [23, 24]. The drug cytotoxicity seems to be the result of the DNA damage caused by breaks during DNA synthesis inhibition, which explains its antineoplastic and teratogenic activity. Nonetheless, HU inhibition of DNA replication is reversible, indicating that the drug is likely a cytostatic agent [6]. Indeed, this agent inhibits DNA synthesis in several organisms and in vitro culture cells; thus, it is mainly active in the S phase of the cell cycle, and the reversibility of its action serves as a cell cycle synchronizing agent in cell cultures [25–28].

Mechanistically, the ribonucleotide reductase (RNR), also known as ribonucleoside diphosphate reductase, is a well-established primary cellular target of HU (Figure 1). RNR is an iron-dependent tightly regulated enzyme that catalyzes the reduction of ribonucleoside diphosphates to deoxyribonucleotide (dNTP) precursors for de novo DNA replication and DNA repair [29–31]. Three main classes of RNRs have been described according to their metallocofactor requirements. In eukaryotes and eubacteria, class I RNRs are oxygen-dependent and contain a dinuclear metal cluster (Fe or Mn); the other classes II and III are found in aerobic and anaerobic microbes that require a cobalt-containing cobalamin (vitamin B_12_) cofactor and a [4Fe-4S]^2+/1+^ cluster coupled to *S*-adenosylmethionine (SAM) for catalytic activity, respectively [32]. Particularly, the mammalian RNR consists of two subunits, *α* and *β*, that can associate to form a heterodimeric tetramer, while the human genome encodes one *α* (RRM1) and two *β*s (RRM2 and RRM2B) [33]. The *α* subunit contains binding domains for ribonucleotide substrates (NDPs/NTPs) and allosteric effectors, consequently regulating the RNR complex by nucleotide pools. In contrast, the *β* subunit possesses catalytic activity and consists of a tyrosyl free radical stabilized by a nonheme iron center necessary for catalysis.

Moreover, the low cell capacity for RNR protein biosynthesis is the rate-limiting step in the de novo synthesis of DNA [30, 34]. Since this enzyme catalyzes the rate-limiting step for DNA biosynthesis, its activity is fine-tuned to generate a periodic fluctuation of dNTP concentration during cell proliferation. In addition, maximum enzyme activity and RRM1 and RRM2 mRNA expression are observed in the S phase of the cell cycle where dNTPs are required [35, 36]. Conversely, at the G_0_/G_1_ phase, the RNR activity is downregulated due to RRM2 gene transcriptional repression, and in the M cell cycle phase, the *β* subunit is subjected to degradation pathways by the anaphase-promoting complex Cdh1 binding and consequent polyubiquitination [37, 38].

HU inhibits the RNR activity in vitro and in vivo, and the duration of DNA synthesis inhibition correlates with the level of deoxyribonucleotide pool reduction [39]. For RNR inhibition, HU, due to its small molecule size, penetrates the RRM2 subunit to directly reduce the diferric tyrosyl radical center via a one-electron transfer mechanism [40–44]. Interestingly, the electron transfer from HU to the tyrosyl radical may be mediated by the generation of nitric oxide-like radicals via H_2_O_2_-dependent peroxidation resulting from the reaction between this agent and the *β* subunits [44, 45].

Because of the inhibition of RNR enzymatic activity by HU, a reduction of the conversion of ribonucleotides to dNTP occurs, and the consequent dNTP depletion leads to an increase in DNA single-strand breaks [46, 47]. Moreover, the depletion of dNTP pools depends on the exposure length and drug concentration for the treatment [48, 49]. The cell arrest in the S phase due to HU-induced dNTP pool reduction slows down DNA polymerase movement at replication forks, which, in eukaryotes, activates the S-phase checkpoint (also called the replication checkpoint kinase pathway). The S-phase checkpoint is a highly conserved intracellular signaling pathway crucial for the maintenance of genome stability under replication stress. In fact, the S-phase checkpoint preserves the functionality and structure of stalled DNA replication forks and prevents chromosome fragmentation [50–52]. When the S-phase checkpoint is activated, it stimulates RNR activity by increasing RNR *β* subunit production and regulating its subcellular localization, while the RNR small inhibitor protein expression is downregulated. Furthermore, the activated S-phase checkpoint delays mitosis, suppresses the firing of late origin, and stabilizes the slowed replication forks against collapse, and this allows for the recovery of the regular DNA synthesis rate when the HU effect diminishes [51–54].

Because of low RNR activity, the deprivation of the dNTP pool below the threshold required to sustain DNA replication fork progression may provoke DNA replication fork collapse, which generates strand breaks and oxidative stress. In addition, HU can provoke direct DNA damage at thymine and cytosine residues in vitro, probably because of the Cu(II)-mediated generation of nitric oxide and H_2_O_2_ [55]. Therefore, these HU's functions may directly cause the permanent effects observed in several cells and discussed later in the text [56, 57].

Even though HU inhibits the RNR activity, which is high in proliferating cells, cells can progress from G_1_ to the S phase at a relatively standard rate, where the drug promotes an accumulation of cells at the early S phase. Consequently, HU reduces the replication fork progression and DNA replication rate [54, 58]. HU selectively eliminates cells in the S phase of highly proliferative cells that are most sensitive to the drug; as mentioned above, HU cytotoxic effects also depend on the dose and duration of exposure [39]. Besides specifically inhibiting RNR, HU also exerts other inhibitory functions on the replitase complex in the S phase of the cell cycle; replitase is a multienzyme complex of mammalian cells that produce dNTPs and deliver them to DNA synthesis by the DNA polymerase. Replitase complex comprises thymidine kinase, dihydrofolate reductase, nucleoside-5′-phosphate kinase, thymidylate synthase, and RNR itself [59, 60].

## 3. Mechanism of Cellular Senescence

Cellular senescence, defined as a process that causes an irreversible proliferative cell arrest with secretory features in response to several molecular and biological stressors, is a significant contributor to aging and age-related diseases [61–64]. This process was initially described by Hayflick and Moorhead in 1961 [65] when they observed that primary cells undergo a limited number of cell divisions in vitro. This observation allows suggesting a cell-autonomous theory of aging that implies the depletion of active replicative cells required for tissue homeostasis and tissue repair and regenerative processes [62].

**Figure 2:**
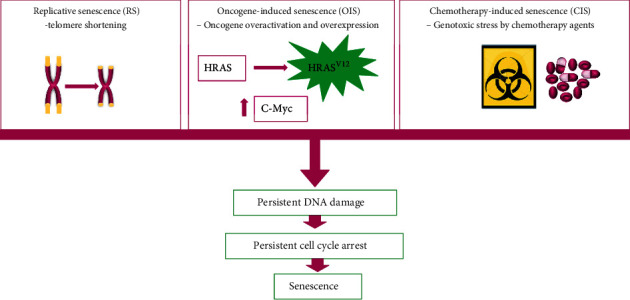
Mechanism of cellular senescence. The figure illustrates the main three senescence types that influence tumorigenesis: replicative senescence (RS) due to telomere shortening from a limited number of cell divisions, oncogene-induced senescence (OIS) due to an aberrant and sustained antiproliferative response to oncogenic signaling resulting from an oncogene-activating mutation and expression or the inactivation of a tumor-suppressor gene, and chemotherapy-induced senescence (CIS) due to cell response to severe genotoxic stress from exposure to a variety of onco-therapeutic agents.

Cellular senescence encompasses different biological and molecular events that result in at least three senescence types (Figure 2): In replicative senescence (RS), the main mechanism relies on the number of cellular divisions in culture in vitro and, consequently, telomere shortening due to successive cell duplication [65–68]. Oncogene-induced senescence (OIS) is related to a tumor-suppressive mechanism as a response to oncogene overactivation and overexpression. Oncogenic activation seems to induce a stable growth arrest in premalignant cells from senescence expression, allowing a blockade of genetically unstable cells to progress to dangerous malignant stages. For instance, H-RAS mediates the induction of cell cycle inhibitor p16^INK4A^, which precludes the hyperphosphorylation of RB by the cyclin-D- and CDK4 and suppresses E2F activity. In addition, increased c-Myc expression promotes the p14^ARF^ transcription that stabilizes p53, thus accelerating cellular senescence [69–72]. The cellular senescence induced by oncological agents used at relevant therapeutic concentrations is called chemotherapy-induced senescence (CIS) [73].

**Figure 3:**
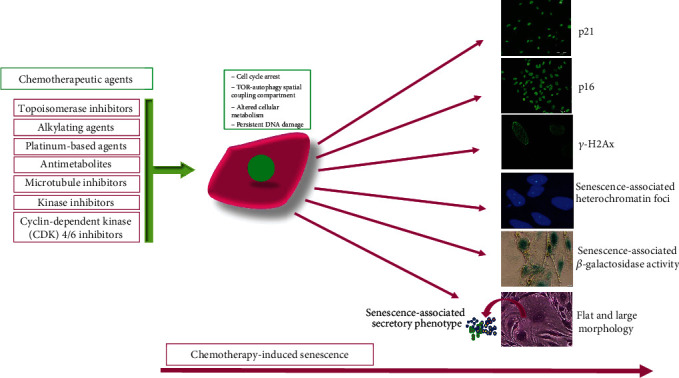
Chemotherapy-induced senescence. The figure indicates the main types of chemotherapeutic drugs with different mechanisms of action that induce genotoxic stress, triggering several cellular and molecular changes that result in the acquisition of senescence phenotype features indicated in the figure, such as increased p21^Cip1^, p16^INK4^, and *γ*-H2Ax expression, senescence-associated heterochromatin foci formation, expression and activity of senescence-associated *β*-galactosidase, senescence-associated secretory phenotype, and morphology changes in flat and enlarger cells.

In this last context, “immortal” cancer cells can undergo senescence from exposure to chemotherapeutic agents, causing severe cellular stress and displaying both protumorigenic and antitumorigenic functions [74, 75]. The chemotherapeutic armamentarium comprises genotoxic and cytotoxic drugs that target proliferating cells in a variety of cell cycle-dependent mechanisms (Figure 3) [76]. These drugs include topoisomerase inhibitors such as doxorubicin, etoposide, and topotecan [77–80]; alkylating agents such as busulfan, cyclophosphamide, and mitomycin C [81–83]; platinum-based agents, including cisplatin, carboplatin, oxaliplatin [84–86]; antimetabolites such as methotrexate, gemcitabine, 5-fluorouracil, and hydroxyurea [87–90]; microtubule inhibitors that comprise paclitaxel, vincristine, and vinblastine [91–93]; kinase inhibitors such as vemurafenib, dasatinib, and lapatinib [94–96]; and cyclin-dependent kinase (CDK) 4/6 inhibitors, including palbociclib, abemaciclib, and ribociclib [97–99].

Interestingly, besides altering cellular cancer states, CIS also affects the tumor microenvironment by acting on noncancerous tissues and promoting immunosurveillance to eliminate tumor cells, while it also may contribute to chronic inflammation and cancer drug resistance [74, 100–102].

With senescence induction, cells display a stable cell cycle arrest and complex phenotypic and molecular changes, such as cell enlargement and flattening, altered cellular metabolism, and dysfunctional mitochondria, and the generation of the cytoplasmic target of rapamycin- (TOR-) autophagy spatial coupling compartment (TASCC) (Figure 3) [103, 104]. Moreover, senescent cells exhibit increased expression and activity of senescence-associated *β*-galactosidase (SA-*β*-gal), a lysosomal enzyme that in senescence conditions stains positive at pH 6 and is one of the first characteristic molecular markers for senescence identification (Figure 3) [105]. Furthermore, because of the inherent molecular changes during the display of senescence features, cells suffer persistent damage such as DNA double-strand breaks that triggers a persistent DNA damage response (DDR), resulting in permanent cell cycle arrest [106]. Specifically, DDR is a signaling cascade network that senses and repairs DNA lesions, thus preserving DNA integrity and preventing the generation of undesirable deleterious mutations, which under persistent or unrepairable DNA damage may drive cells toward apoptosis or cellular senescence [107]. In this sense, in higher organisms, the DDR prevents neoplastic transformation, ensuring the termination of cellular proliferation and the removal of severely damaged cells [108].

Cells may display senescence-associated heterochromatin foci (SAHF), detectable with immunostaining techniques (Figure 3), which result from the association of the retinoblastoma (Rb) tumor suppressor and heterochromatin protein (HP) 1, DNA methyltransferase (DNMT) 1, or the suppressor of variegation 3–9 (Suv39) methyltransferase, which together form repressive complexes for the E2 transcription factor (E2F) 1 gene targets [109]. Moreover, the DNA damage caused by senescence inducers provokes the formation of persistent nuclear foci or DNA-SCARS characterized by chromatin alterations that reinforce cellular senescence [110]. In classical or normal reparative conditions, this process forms early foci that can be detected by *γ*-H2Ax or 53BP1 staining; in successful normal DNA repair, their expression rapidly disappears, while in senescence, these structures persist longer because of the elevated damage to the DNA, thus allowing the DNA-SCARS formation [111]. Moreover, DNA damage is sensed by ataxia telangiectasia-mutated (ATM), an essential response kinase coordinating checkpoint, and senescence responses. ATM is activated by either DNA breaks or oxidative stress and plays an essential role in the senescence response by phosphorylating and stabilizing p53 [112–116]. From a molecular viewpoint (Figure 3), the upregulations of the tumor suppressor Rb-p16^INK4A^ and p53-P21^Cip1^ pathways (Figure 3) are molecular hallmarks that participate in the induction of cellular senescence by downregulating cyclin/CDK and inhibiting E2F1 activity [62, 117]. In addition, downregulation of the nuclear lamina protein lamin B1 has also been postulated as a feature of the senescent phenotype [118, 119].

Even though cellular senescence implies a permanent cell cycle arrest, these cells remain metabolically active, earning the nickname “zombie” cells, and interact with other cells in the tumor microenvironment by cell-cell interaction or via the senescence-associated secretory phenotype (SASP), influencing the fate of neighboring cells via bystander effects (Figure 3) [120, 121]. The SASP encompasses a plethora of cytokines, growth factors, and proteases such as interleukin- (IL-) 1, IL-6, IL-8, growth-regulated oncogene (GRO) *α*/*β*, granulocyte-macrophage colony-stimulating factor (GM-CSF), insulin-like growth factor binding proteins (IGFBPs), matrix metalloproteinases- (MMP-) 1, MMP-3, and MMP-10, intercellular adhesion molecule- (ICAM-) 1, and plasminogen activator inhibitor type 1 (PAI-1) [122, 123].

Nevertheless, a significant challenge is to typify senescence cells accurately. None of the above markers can be considered universal, and typifying senescence requires different phenotypical, biochemical, and molecular measurements. Recently, a combination of cytoplasmic markers, such as SA-*β*-gal, proliferation markers that are nuclear-localized, including p16^INK4AA^, p21^WAF1/Cip1^, Ki67, and SASP expression, have been recommended to standardize senescence characterization (Figure 3) [61].

Although CIS often is associated with tumor growth inhibition and regression [74], senescent cells may remain after the termination of onco-therapies and promote tumor progression by the SASP because they promote tumor cell dormancy, therapy resistance, and cancer relapse [64, 124–128]. In addition, SASPs influence the progression of surrounding nonsenescent tumor cells and metastasis by influencing the tumor microenvironment by factors that may promote the epithelial-to-mesenchymal transition (EMT), thus accelerating migration, invasion, and cancer cell malignancy features [129–132].

## 4. Cellular Oxidative Stress and Hydroxyurea

Reactive oxygen species (ROS) are constantly generated in normal physiological conditions, and they are eliminated by scavenging systems, thus maintaining cellular REDOX homeostasis. Meanwhile, dysbalance of this homeostasis due to aberrant ROS production or antioxidant decrease contributes to tumor progression and is a hallmark of several types of cancer (Table 1) [133, 134]. Moreover, exacerbated ROS levels result in biomacromolecular damage of proteins, lipids, and DNA among others, which promotes cellular senescence and aging and is associated with the physiopathology of several age-associated diseases [135].

ROS comprise a family of highly reactive molecules that regulate normal cellular conditions by fine control of the generation/consuming rate. In contrast, in cancer, a dysregulated oxidative stress is produced that contributes to the chemical damage of proteins, lipids, and DNA and tumorigenesis promotion [136]. From a molecular viewpoint, ROS are small molecules derived from the oxygen comprising free radical and nonfree radical oxygen intermediates, ions, or molecules that have a single unpaired electron in their outermost shell of electrons. Moreover, ROS are constantly generated inside cells by enzyme complexes or as by-products of REDOX reactions, including those underlying mitochondrial respiration [137, 138]. These molecules include oxygen radicals, such as superoxide anion, hydroxyl, peroxyl, and alkoxyl, and nonradical molecules that are either oxidizing agents or easily converted into radicals, such as hypochlorous acid, ozone, singlet oxygen, and hydrogen peroxide. In addition, this oxygen-containing reactive species can combine with nitrogen to generate nitrogen-containing oxidants such as nitric oxide and peroxynitrite that belong to the family of reactive nitrogen species (RNS) [136, 138]. Furthermore, the REDOX dysbalance in cancer cells is generated by increased cellular metabolic activity, mitochondrial dysfunction, deregulated cellular receptor signaling, peroxisome activity, oncogene activation, cyclooxygenase lipoxygenases, and thymidine phosphorylase. In addition, the contribution to the REDOX dysbalance of these factors may depend on the malignant stage of the cancer cells and their interaction with tumor stroma and infiltrating immune cells [139, 140]. Furthermore, cellular superoxide anions form mainly because of the NADPH oxidase (NOX) family [141]. Five forms of NOXs have been found: the small GTPase Rac1-dependent NOX1, NOX2, and NOX3, and the small GTPase Rac1-independent NOX4 and NOX5 [142].

ROS participate in different aspects of tumor development and progression; they regulate intracellular signaling pathways involved in cell proliferation and survival while also influencing cell motility, invasiveness, and metastasis and regulating inflammatory responses within the tumor stroma and in angiogenesis [140]. Furthermore, ROS contribute to determining mammalian cells' senescent cellular fate [143, 144]. These oxygen-containing reactive species can promote cellular senescence by telomere-dependent mechanisms and telomere-independent mechanisms involving unrepairable single or double-strand DNA breaks [145, 146]. Moreover, their excessive levels generate DNA lesions by forming 8-oxo-2′-deoxyguanosine, which accumulates in senescent human cell cultures and aging mice. Consequently, this DNA damage generates genomic instability, DNA mutations, and tumor development [147]. Therefore, ROS produce genomic alterations such as point mutations and deletions, which may inhibit tumor-suppressor genes while activating and inducing the expression of oncogenes to further contribute to the enhancement of cancer cell malignancy [143].

On the other hand, ROS also regulates cellular proliferation, which depends on their levels and duration of exposure. In this sense, most cytostatic/cytotoxic anticancer drugs inhibit cancer cell proliferation and cell survival by promoting ROS generation [148, 149]. For instance, both H_2_O_2_ and its dismutation product superoxide (O_2_·^−^) reduce cancer cell proliferation, while H_2_O_2_ may also form, via Fenton reaction, the hydroxyl radical (OH·) that highly inhibits cell proliferation [149].

Although HU can enhance cellular oxidative stress, the intimate molecular mechanism is not well understood. Some earlier studies have suggested that this drug may exert cytotoxic effects through radical chain reactions via H_2_O_2_ and initiated by its hydroxylamine group. Conversely, radical scavengers substantially reduce the cytotoxic and teratogenic HU activities [150, 151]. Moreover, HU causes DNA damage to thymidine and cytosine residues via increasing H_2_O_2_, in part by inducing ROS via provoking a fork collapse. Moreover, this agent induces mutagenic DNA lesions in V79 Chinese hamster cells, likely due to the generation of H_2_O_2_ [6, 45, 57, 152].

Moreover, nitric oxide radical (·NO), generated upon the 3-electron oxidation of the drug, may be responsible for many of its pharmacologic effects, including the RNR enzyme inhibition [153, 154]. Nevertheless, recent analyses indicated that HU might downregulate the expression of scavenger proteins, such as superoxide dismutase (SOD) 2 and peroxiredoxin-1 (PRDX1), and regulatory oxidative stress proteins, such as Sirtuin- (Sirt-) 3 (Table 1) [154–156]. Although the involved molecular mechanisms by which HU regulates the expression of these proteins have not been well elucidated so far, the induced deficiency of these oxidative stress regulatory proteins significantly contributes to the elevation of ROS by HU and the establishment of cellular senescence.

## 5. Hydroxyurea and Cellular Senescence

**Figure 4:**
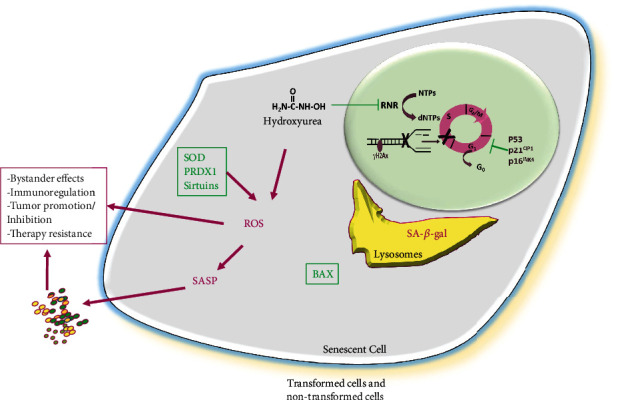
Overview of the main features of hydroxyurea-induced cellular senescence. Hydroxyurea, by inhibition of ribonucleotide reductase (RNR), dramatically reduces the synthesis of deoxyribonucleotides (dNTPs) from ribonucleotide substrates (NTPs). This dNTP pool reduction provokes a termination of DNA replication and may result in replication fork collapse. Furthermore, because of genotoxic HU action, DNA damage is generated, and phosphorylated histone H2AX (*γ*H2AX) binding to DNA breaks is promoted. Cells may suffer an arrest at the S cell cycle phase, concomitant with increased expression of cell cycle inhibitors p16^INK4A^, p21^Cip1^, and p53, reinforcing the cell cycle inhibition. During senescence induction, cell size is enlarged, and lysosomal biogenesis is increased, as indicated by elevated levels of expression and senescence-associated-*β*-galactosidase (SA-*β*-gal). Along with DNA replication inhibition, augmentation of oxidative stress occurs as reactive oxygen species (ROS) expression levels are elevated, consistently reducing antioxidative stress protein superoxide dismutase (SOD) 2, peroxiredoxin (PRDX) 1, and Sirtuins that contribute to maintaining increased oxidative stress. Moreover, HU-induced senescent cells are refractory to apoptosis, in part from reduced expression of the proapoptotic BAX protein. Senescent cells are metabolically active, and they express and release a set of factors as part of the senescence-associated secretory phenotype (SASP). The SASP may profoundly influence surrounding cells and tissues through increased local and systemic inflammation and regulation of immune response, depending on SASP pattern, positively or negatively affecting tumor growth, and may also contribute to therapy resistance. Magenta words mean increased expression. Magenta arrows mean induction. Gree T-shape symbols mean inhibition. Green words mean reduced expression.

HU inhibits proliferation in several organisms and cell lines. At therapeutically relevant levels, HU mainly induces cell proliferation arrest in the S cell cycle phase because of the decrease in dNTPs by RNR enzymatic activity inhibition [157, 158]; this causes a reduction of DNA polymerase movement at replication forks that generate a DNA replication stress [6, 102]. In cancer therapy, this agent is frequently used as an antitumor agent because of its cytoreduction functions. Moreover, HU belongs to the family of antimetabolite drugs that can induce premature cellular senescence from interfering with the crucial synthesis pathways required for DNA duplication (Figure 4) [102, 128].

One of the first observations that HU may promote senescence-like phenotype in cancer cells was made in the human erythroleukemia K562 cell line. K562 cells underwent cell proliferation arrest and positivity to SA-*β*-gal activity after seven days of HU treatment. Moreover, the treatment increased the expression of the cyclin-dependent kinase inhibitors p16^INK4A^ and p21^Cip1^ [159]. Interestingly, since K562 cells are p53-deficient [160], HU-induced senescence can occur independently of p53 activity in these cells. Additionally, this agent also induces cellular senescence in rat hepatoma McA-RH7777 cells; after treatment, cells exhibited enlarged size, increased SA-*β*-gal positive staining, and a substantial reduction in cell proliferation as cells were arrested in the G_0_/G_1_ cell cycle phase. In this case, a substantial reduction in the cellular frequency at the G_2_/M phase was observed. Cells undergoing HU treatment consistently expressed elevated levels of p21^Cip1^ associated with cell cycle arrest at the G_1_/S interphase [161]. Likewise, the drug promotes cellular senescence in neuroblastoma cell lines after a relatively long period of treatment, in part because of HU concentrations below 200 *μ*M. After five weeks of treatment, more than 50% of the cells stained positive for SA-*β*-gal, and in this period, cells exhibited a reduction of telomere length that was 50% of the cells after ten weeks [162]. Although this pharmaceutical compound induces neuroblastoma cell senescence in vitro, it does not promote cell secretion of unfavorable SASPs, such as MMP-9, the monocyte-chemotactic protein- (MCP-) 3, the regulated-on activation normal T cell expressed and secreted (RANTES), and the vascular endothelial growth factor (VEGF). In contrast, it induces secretion of IL-6 and platelet-derived growth factor- (PDGF-) AA, involved in immuno-regulation and angiogenesis [80, 163–165].

Besides cancer cells, HU may affect nontransformed cells. For instance, in a model of foreskin fibroblast cells, treatment with the drug in the range of 400–800 *μ*M provoked a reduction of cell proliferation and morphological changes similar to the findings in replicative cellular senescence; moreover, these changes were not reversible by removing the drug treatment. HU treatment induces SA-*β*-gal activity and p53 and p21^Cip1^ expression along with Jun N-terminal kinase (JNK) activation. Moreover, because of HU treatment, senescence fibroblasts are protected from UV light-induced apoptosis [166]. Similar results were reported in a human embryonic fibroblast cell line; the treatment with this medical agent induced SA-*β*-gal and p21^Cip1^; moreover, the elevated p21^Cip1^ expression seemed due to increased protein stability rather than de novo synthesis. In addition, increased p21^Cip1^ was independent of increased p53; thus, suggesting that in these cells, p53 activity was not implicated [167], which is concordant with the theory that p53 mainly transcriptionally activates p21^Cip1^ expression [168]. In addition, the HU-induced senescence in mouse fibroblasts, determined by SA-*β*-gal activity, is increased by transcription factor c-Jun depletion, while c-Jun overexpression inhibits the senescence induced by the treatment and drives cells to cell death.

Meanwhile, the transcription factor JunB enhances HU-induced senescence by upregulation of their direct target p16^INK4A^. These results suggest that the balance between the c-Jun and JunB transcription factors may determine the cellular response to the chemotherapeutic HU agent [169]. In addition, the chronic exposure of rat and human fibroblasts to low concentrations of the chemotherapeutic agent induced cellular senescence by a p53-dependent p21^Cip1^ expression and increased SA-*β*-al activity, but independent of p16^INK4A^. Moreover, HU induces reversible *γ*H2A.X foci, indicating that replicational stress induced by HU promotes DNA strand breaks [58].

HU treatment also can induce postnatal subventricle neural stem cells (NSCs) to undergo cellular senescence [154]. In this case, elevated concentrations of the drug (at mM levels) cause persistent DNA damage evidenced by *γ*H2AX foci formation and a consistently increasing number of SA-*β*-gal positive cells, as well as increased p16^INK4A^, p21^Cip1^, and p53 expression. Moreover, under HU treatment, cells suffered a reduction of proliferation as a consequence of a cell cycle arrest at G_0_/G_1_. Furthermore, the treatment increased intracellular ROS levels along with a significant decrease in SOD2 and PRDX1. SOD2 is a main antioxidant enzyme that scavenges ROS in the inner mitochondrial matrix and acts as the first defense against mitochondrial oxidative stress [170], while PRDX1 is a thiol-specific peroxidase that scavenges hydrogen peroxide [171]. In addition, this pharmaceutical agent provokes a downregulation of Bcl-2-associated X protein (BAX), a critical proapoptotic factor that may contribute to the decreased apoptosis observed in senescent NSCs [154, 172]. In addition, HU-induced NSC cellular senescence is counteracted by *α*-glycerylphosphorylethanolamine (GPE) [173], which is a precursor biomolecule of phospholipid synthesis and exerts neuroprotective effects in human hippocampal cells [174]. For instance, GPE protects NSCs from the induction of DNA damage caused by phosphorylated *γ*H2AX levels and rescues cell proliferation from HU inhibition. Furthermore, GPE highly reduces HU-induced SA-*β*-gal expression and activity and p53 and p21^Cip1^ mRNA expression. Moreover, this chemotherapeutic agent increases the ADP/ATP ratio that indicates mitochondrial energy metabolism impairment, while GPE restores the physiological ADP/ATP ratio and significantly reduces HU-induced ROS levels. GPE also consistently inhibits the ROS-responsive NF-*κ*B signaling [175]. Thus, GPE protects NSCs from HU-induced cellular senescence, indicating that it might function as an antiaging compound for NSCs [173].

HU can also induce cellular senescence of mesenchymal stem/stromal cells (MSCs). MSCs are multipotent cells characterized by their ability to differentiate into adipocytes, chondrocytes, and osteoblasts; their expression of surface markers CD73, CD90, and CD105; and their lack of hematopoietic lineage markers [176, 177]. They are also present in the tumor microenvironment, where they support the growth of tumor cells, activate mitogen and stress signaling, and increase resistance to cytotoxins [178, 179]. HU at relatively high levels inhibits dental follicle-derived MSC proliferation and clone formation capacity along with increased DNA double-strand breaks indicated by *γ*H2AX foci formation; additionally, it induces SA-*β*-gal activity and a higher expression level of p53, p21^Cip1^, and p16^INK4A^. These effects are accompanied by reducing MSC differentiation toward adipogenic, chondrogenic, and osteogenic lineages.

Moreover, senescence induction by HU increases ROS levels along with the downregulation of SOD2 [155]. Similarly, peripheral blood MSCs (PB-MSCs) are also targeted by this agent [180]. HU induces a senescence-like phenotype in PB-MSC as it provokes substantial cell morphology changes accompanied by SA-*β*-gal and p16^INK4A^ expression with a discrete effect on p21^Cip1^ expression. The treatment with the drug at therapeutically relevant concentrations (200 *μ*M) strongly induces cell cycle arrest to the S cell cycle phase; consistent with that, in the presence of HU, cells progress from G_1_ to the S phase at a normal rate and are arrested in the early S phase [58]. This pharmaceutical compound also increases intracellular ROS levels that contribute to senescence induction because oxidative stress scavengers, N-acetylcysteine, and NOX inhibitor apocynin inhibit cellular senescence and partially protect PB-MSC proliferation from inhibition by HU. Furthermore, HU-induced senescent PB-MSCs significantly inhibit the proliferation of erythroleukemia cells by secreting TGF-*β*1 and elevated ROS production. Thus, senescent PB-MSCs may shift from a tumor-promoter activity to a tumor-suppressive function [180].

As stated, HU during senescence induction promotes an elevation of cellular ROS in part because of downregulation of SOD2, and recently, it was reported that this drug could also inhibit the expression of Sirt-3 (Figure 4) [156]. Sirt-3 is a mitochondrial deacetylase that regulates major mitochondrial biological processes, including ATP generation, ROS detoxification, nutrient oxidation, mitochondrial dynamics, and the unfolded protein response [181, 182]. Sirt-3 also deacetylates and thereby activates SOD-2 [183]. HU induces mouse embryonic fibroblast (MEF) senescence and increases ROS levels and Sirt-3 and SOD2 downregulation. Interestingly, adjudin is a compound derived from the anticancer drug lonidamine that acts through Sirt-3 activation [184]. Adjudin delays HU-induced cellular senescence reducing ROS levels by Sirt-3 upregulation [156]. Although it reduces the anti-ROS proteins Sirt-3 and SOD-2 expression during cell senescence induction, no molecular mechanism implicated in their downregulation has yet been elucidated. Nevertheless, it is important to reveal the underlying mechanistic pathways of elevated ROS levels due to HU treatment. Moreover, adjudin, due to its antisenescence function, may contribute to the therapy for age-associated diseases and CIS.

Similarly, 1,5-isoquinolinediol (IQD), a poly (ADP-ribose) polymerase (PARP1) inhibitor, protects MEF cells from HU-induced senescence [185]. PARPs perform poly(ADP-ribosyl)ation of proteins as an immediate cellular response to genotoxic insults induced by ionizing radiation, alkylating agents, and oxidative stress [186]. HU accelerates the MEF replicative senescence rate by inducing oxidative stress paralleled to increasing PARP1 and lamin A expression, while IQD effectively suppresses the senescence rate by decreasing the activity of PARP1 [185]. Noticeably, the increased expression and activity of PARP1 rapidly consume the NAD+ necessary for Sirt-1 function, so the decreased Sirt-1 activity results in increased oxidative stress. Thus, pharmacological PARP1 inhibition may restore NAD+ levels and Sirt-1 activity and normalize oxidative metabolism [187], which may help control the prosenescence function of HU and prevent chemotherapy-associated accelerated aging in cancer survivors [188].

## 6. Concluding Remarks

HU as a nonalkylating antiproliferative agent is still used to manage a variety of disease conditions in both neoplastic and nonneoplastic settings, and it is listed as an essential medicine by WHO. This drug can function as a cytoreductive agent because of its cytostatic properties; in this sense, as is analyzed in this review, HU can induce cellular senescence in both cancer cells and nontransformed cells, which profoundly affects tumor growth and homeostatic function of normal cells. Mechanistically, this compound functions as an antimetabolite agent by acting on RNR and affecting the generation of the dNTP pools necessary for DNA synthesis and duplication. The dNTP deficiency may cause fork collapse associated with DNA damage and ROS generation, which contributes to establishing a cellular senescence phenotype. What is the molecular mechanism by which HU increases ROS? It is a relevant question to address experimentally; cells under treatment may exhibit reduced expression of antioxidative stress, SOD2, PRDX1, and Sirtuins that contribute to the enhancement and stabilization of elevated ROS levels. For instance, repression of SOD2 may occur at the level of epigenetic regulation [189], and HU may promote epigenetic modifications along with regulation of several intracellular signal transductions, such as MAPK, PKG, and PKA signaling [190], which, in part, may explain the reduced expression of SOD2 during the increase in ROS levels and the cellular senescence due to HU treatment.

Different strategies have emerged to eliminate CIS cells because of the need to eliminate tumor cells and nontransformed dysfunctional cells. To this end, senolytic strategies have been developed to target CIS-transformed cells and, potentially, the nontransformed senescent cells without affecting normal proliferating cells [191]. In addition, the increased ROS levels that contribute to HU-induced cellular senescence are valuable targets for developing therapeutic strategies to improve the cytotoxic function of the drug, which may shift cells from the senescence response toward cell death fate [192]. Understanding the delicate balance between cellular senescence and the beneficial anticancer function of HU is vital to improving the current therapies to impact the life quality of patients and control the undesirable premature aging caused by chemotherapy.

## Figures and Tables

**Table 1:** Reactive oxygen species and hydroxyurea main functions and effects on tumorigenesis.

Function	Cellular and molecular effects	Ref.
*Reactive oxygen species*		
Intracellular signaling pathway regulation	Cell proliferation and survival, cell motility, invasiveness, and metastasis	[140]
Senescence induction	Telomere-dependent mechanism and telomere-independent mechanism (i) Double-strand DNA breaks induction (ii) DNA lesions due to 8-oxo-2′-deoxyguanosine generation (iii) Genomic instability (iv) Gene mutations implicated in the following: (a) Inhibition of tumor suppressor genes (b) Activation of oncogenes	[143–147]
Regulation of cellular proliferation	H_2_O_2_, superoxide (O_2_·^−^), and hydroxyl radical (OH·) reduce cell proliferation	[148, 149]
*Hydroxyurea and reactive oxygen species*		
Cytotoxicity	Cytotoxicity and teratogenicity due to radical chain reactions, via H_2_O_2_, initiated by HU hydroxylamine group to form R-HṄOH^+^ radical and generation of NO	[150, 151]
DNA damage by increasing oxidative stress	Thymidine and cytosine damage via increasing NO and H_2_O_2_ and fork collapse	[6, 45, 55, 152]
Nitric oxide generation	RNR enzyme inhibition via NO and nitrosyl radical ·NO production	[45, 153, 154]
Scavenger protein inhibition	Downregulation of superoxide dismutase-2, peroxiredoxin-1, and Sirtuins	[154–156]

## Data Availability

No datasets were generated or analyzed during the current study.
